# Conceptualizing participation in the community mental health context: Beginning with the Clubhouse model

**DOI:** 10.1080/17482631.2021.1950890

**Published:** 2021-07-12

**Authors:** Kimiko Tanaka, Eric Stein, Thomas J Craig, Liv Grethe Kinn, Julie Williams

**Affiliations:** a Department of Social Welfare, Tokyo Fukushi University, Tokyo Japan; b Center for Social Work Education, Widener University, Chester, PA, U.S.A; c Health Services and Population Research Department, Institute of Psychiatry, Psychology & Neuroscience, King’s College London, London, UK; d Department of Health and Social Sciences, Western Norway University of Applied Sciences, Bergen, Norway

**Keywords:** Conceptualization, participation, community mental health, Clubhouse, decision-making, work activity, Community, autonomy, egalitarian connection, well-being

## Abstract

**Purpose:**

Although participation is key to community mental health, the concept remains elusive. The study explored a conceptualization of participation in the community-based mental health agencies context from a first-person perspective, using the Clubhouse model as an example.

**Methods:**

Qualitative data, collected from 21 Clubhouse service users through three focus groups (1 UK and 2 US) for primary analysis and secondary data from 104 individual interviews, were analysed using a grounded theory approach.

**Results:**

Focus group narratives revealed three main domains of what may be named everyday participation process, Making Decisions, Doing Work, and Locating Oneself in Community, blended with each other rather than forming clear-cut stages. Sixty-six extracted primary codes, with two underlying interrelated core categories identified, named Autonomy and Egalitarian Connection, were organized by domain and by category.

**Conclusions:**

The findings suggest a 3 × 2 axial model of participation that participation signifies a behaviour, comprised of three blended activity domains, entailing actions and interactions that concern Autonomy and Egalitarian Connection, which, dynamically interacting with each other, appear to condition meaningful participation the next day. Egalitarian relationship skills development appears critical for training practitioners to help promote service users’ quality everyday participation and getting-a-life-back experiences towards well-being, or meaningful life.

## Introduction

Despite the centrality of participation in the field of community mental health (CMH), a definition of the concept has remained somewhat elusive. A significant amount of theoretical or conceptual work on participation exists but it is largely limited to spheres involving the general public, such as political science, urban planning, development, media, or business (Arnstein, 1969; Carpentier, 2011; Cornwall, 2008, 2011; Delwiche & Henderson, 2013; Kelty et al., 2015; May, 2006; Pateman, 1970; Stage & Ingerslev, 2015; WHO, 1991). Only recently have studies targeting specific minority populations been emerging, such as persons with physical disabilities (Dijkers, 2010; Ginis et al., 2017; Law, 2002; Stallinga et al., 2014). In the CMH context, Salzer and his associates (Salzer et al., 2014, 2015; Wong et al., 2007) have advanced a domain-specific conceptualization that is fairly reduced to objective indicators of participation. A few qualitative studies have focused on the subjective experiences of participation in activities occurring in community life, which are either descriptive (Schiff et al., 2008; Yilmaz et al., 2008, 2009) or based on staff views (F. P. Chen & Oh, 2019).

The purpose of the present study was to explore a conceptualization of participation in the CMH context from a “Nothing about us without us” (Charlton, 2000), or first-person perspective. We began this work by examining the mental health Clubhouse model, which is purported to emphasize participation (J. Lanoil, Clubhouse consultant, personal communication, n.d.) and the term participation is widely used in the Clubhouse literature including in titles (Anderson, 1999; Beard et al., 1982; Carolan et al., 2011; Clubhouse International, 2020, 2021; Doyle et al., 2013; Herman et al., 2005; Norman, 2006; F. P. Chen & Oh, 2019; Pernice et al., 2021; Raeburn et al., 2013, 2015; Schiff et al., 2008; Tanaka & Davidson, 2015a; Tanaka et al., 2018). To our knowledge, however, no study has yet to explicitly theorize on this concept in the Clubhouse context.

### The Clubhouse model

Clubhouse is a non-profit organization that provides psychosocial day services for adults with serious mental illness to assist their lives in the community (Clubhouse International, 2021; Hänninen, 2012). Founded in 1948 in the U.S., the model is operationalized by the International Standards for Clubhouse Programs (Clubhouse International, 2020) and has been internationally disseminated since 1989. Clubhouse International, the model’s certification body, routinely assesses Clubhouse quality using the Standards as a fidelity measure. As of 2021, nearly 260 Clubhouses in over 30 countries are certified, with about 85 additional Clubhouses working towards achieving certification (Clubhouse International, 2021). The Standards (Anderson, 1999; Clubhouse International, 2020) consists of 35 statements constituting seven sections: membership, relationships, work-ordered day, employment, education, functions of the house, and funding, governance and administration. For example, the “Membership” section declares the rights of service users, aka “members,” to “choose the way they utilize the clubhosue and the staff with whom they work” (§3; Clubhouse International, 2020), followed by the statement of no contracts or rules that mandate members’ participation. One of the sections on employment designates “transitional employment” as a members’ right irrespective of work habits, skills, or evaluation by professionals. The “Relationships” section defines the member-staff relationships as “collegial:” “All clubhouse meetings are open to both members and staff. There are no member-only meetings or staff-only meetings where program issues and member issues are discussed” (§20).

Clubhouse underscores work activity participation through its core programs, the work-ordered day and transitional employment (Beard et al., 1982; Clubhouse International, 2020, 2021; Doyle et al., 2013; Hänninen, 2012). Members are encouraged to participate in the work-ordered day during weekdays, which, unlike conventional day treatment programs, is ordered, or structured, around work activities just like businesses. Beginning with work unit meetings, members’ daily work life unfolds within the Clubhouse from 9AM to 5PM. Unlike the competitive world of work, however, the Standards (Clubhouse International, 2020) directs members’ work participation to be strictly voluntary (§3), while facilitating their participation by optimizing the number of Clubhouse staff in a way that is “sufficient to engage the membership, yet few enough to … [necessitate] member involvement” (§9). Within this structure, members and staff collaborate “side-by-side” to achieve tasks that are necessary to operate the Clubhouse (§15). Through the work-ordered day, members’ employment needs are identified and supported through transitional or other employment programs.

### The present study

In the absence of a CMH-specific theory on participation, we took a data-driven approach to the conceptualization of participation (Corbin & Strauss, 2015; Glaser & Strauss, 1967; Kelle, 2014). We first explored and subsequently described members’ understanding and experience of participation as process to answer our research questions posed under a paradigm for grounded theory process framed by Corbin and Strauss (2015): In members’ view, what is participation like at Clubhouse? What kinds of activities does their participation entail and how do these activities unfold? How do they experience these activities? What actions and interactions as well as emotions and thoughts are involved, and under what conditions? What are some of the consequences of their participation? What do these experiences mean to them? We then formulated propositions based on the findings.

## Methods

The study received approval from the ethics committee of the first author’s institution (Tokyo Fukushi University, Tokyo, Japan, ref. 2018–04).

We analysed two sets of qualitative data, primary data collected through focus groups and secondary data from an earlier study comprising individual interviews and participant observation. For both data sets, inclusion criteria were registered members of Clubhouses recognized by Clubhouse International (2021) who were not hospitalized at the time of research administration. With our limited resources, we used what Corbin and Strauss (2015) allowed as an alternative to the standard Grounded Theory Approach (GTA) to sampling and gathered data before formal analysis. We did not collect additional primary data, either, because we confirmed data saturation during our secondary analysis. Purposive sampling was used to select participants who had something to share about their Clubhouse experiences. We considered informed written consent to study participation sufficient for this purpose. All interviews were unstructured, allowing participants to freely talk about their experiences at their own pace, which helped us to explore “the richest source of data for theory building” (Corbin & Strauss, 2015, p. 38). Each interviewer adopted a style that felt natural to each of us, but our topic not being sensitive, we did not get a sense that interviewers’ characteristics, including the first author being a different nationality from that of interviewees’, critically influenced the quality of data (Corbin & Strauss, 2015; Pezalla et al., 2012). All of us, being applied scientists, inherently had a stance that Patton (2014) may call pragmatic and, as CMH experts or generalist social workers, shared the core principle of rapport and professional use of self-disclosure in our identities as helping professionals, a style that is consistent with that widely used for research interviewing (Fontana & Frey, 1994; Patton, 2014; Pezalla et al., 2012). All the sessions were audiotaped, transcribed verbatim, and de-identified for analysis.

For the primary data, a total of 21 Clubhouse members participated in one of three one-hour focus groups (4–10 participants per group; ages 24–68). The data collection method was utilized to explore first-person experiences and multiple perspectives in a time-efficient manner (Gibbs, 1997; Morgan, 1996, 2018). The groups took place between November 2018 and March 2019 at three Clubhouses, two small ones in the U.S. (referred to below as North and South) and one middle-sized in the U.K. We had two Clubhouses recruit participants and one moderator made a study announcement on site at the other Clubhouse. This difference in recruitment method was somewhat unexpected but it seems to have helped increase the diversity of participants’ responses with respect to a continuum of positive-to-negative experiences.

Moderators ran the focus groups at quiet places on site during the work-ordered day. All the moderators were seasoned in research and/or program evaluation as well as in group interviews. Although moderators had no prior knowledge of any participants and vice versa, all the moderators were familiar with the Clubhouse model. One group was moderated by a former Clubhouse staff/director with no history of formal affiliation with the Clubhouses under study; one was conducted by the second author and his university colleague (an advisory board member of the Clubhouse), and one by the third (a patron of the Clubhouse) and last authors.

We had two predetermined open-ended questions (“What has it been like for you to participate in the Clubhouse?” and “How have you been participating in decision-making?”) and, when needed, moderators probed for details in the natural conversational flow. Following the moderator(s)’ lead, the group began with the first question by alternately sharing each participant’s experiences or perspectives as in individual interviewing, with later interactions among participants naturally occurring from time to time. All participants had the chance to give their own in-depth accounts. Some were more talkative or articulate than others; and one person started out somewhat dominantly but not throughout the group discussion. When it was another participant’s turn, he became an audience without interrupting.

The secondary data (*N* = 104; ages 18–69) were used for “theoretical sampling” purposes in the sense of exploring the validity of the themes emerging in our primary data (Corbin & Strauss, 2015). The data had been collected 6–10 years prior as part of a larger study for a different purpose, to understand the nature of the work-ordered day program. These data were gathered from six Clubhouses of various sizes and locations in two leading countries using the model, the U.S. and Finland. The first author and one research assistant per setting recruited participants through verbal and written study announcements and conducted one-hour face-to-face individual qualitative interviewing during the work-ordered day in a private room within the Clubhouse building. The first author, an East Asian national who had lived in the U.S. for more than 15 years, conducted all interviews in English, always together with a native-language speaking research assistant so as not to miss subtleties in language. Eight (out of 24) Finnish participants chose to respond in Finnish and the research assistant interpreted the interviews. To supplement our individual interviewing, we also collected some observational data throughout the data collection by participating in the work-ordered day at the sites whenever possible and later wrote a limited number (10) of field notes (Adler & Frey, 1994; Fetterman, 1998). The first author was familiar with the Clubhouse model, as well as with several interviewees, through a prior year-long weekly-to-monthly volunteer experience at one of the Clubhouses that helped with the study.

### Data analysis

We followed a GTA as a meta-framework for analysis, which would best serve our study purposes: (a) conceptualizing participation as (b) process that is (c) “grounded” in data, or the lives of the population under study (Corbin & Strauss, 2015; Glaser & Strauss, 1967). To analyse participation as process, we used the paradigm for coding proposed by Corbin and Strauss (2015), which comprises of conditions, actions-interactions-emotions, and consequences within a given context. Throughout the analysis, for which we used qualitative computer software ATLAS.ti 8, we primarily relied on *in vivo* codes (Corbin & Strauss, 2015) as a way to stay as grounded in the data as possible. With the first author’s lead, the first two authors conducted primary analysis. As a form of triangulation (Denzin, 2017), the two had weekly zoom meetings to discuss the analysis to reach a consensus and received periodic input from the other authors when needed to obtain their independent views.

We followed the analytical procedure outlined by Corbin and Strauss (2015). First, we read all the focus group transcripts and listened to the tapes to familiarize ourselves with the primary data and get a main idea of what each set of data was about with respect to participation. Second, we divided each transcript by topic while starting open-coding line by line of the U.S. South data, often mulling over until a *concept* emerged of itself and asking what the portion of data might be telling us. Third, with some sense of direction gained, we launched our *micro-analysis*, coding line by line in more detail the remaining transcripts to evaluate our initial concepts by using various techniques such as *constant comparisons, questioning, looking at language*, and *looking for the negative case*, as well as checking our own and participants’ possible biases. We iterated this micro-analysis back and forth under emergent *guiding questions*, repeating line-by-line coding to evaluate still fluid concepts, some of which, while elaborating on with their *properties* and *dimensions*, we grouped and regrouped into *categories*, or *themes*, with tentative names. *Theoretical comparisons* and writing *memos* were particularly helpful to see the forest and untangle the entangled. With time, concepts and properties derived during our early analysis became more coherent and focused.

The final phase was the secondary analysis of the relevant portion of the earlier individual interview data, entailing continual constant comparison to examine the validity of our evolving grounded theory. Due to the first author’s familiarity with the data from her past studies, however, we did this analysis informally without explicit coding (Clarke, 2003) once the data started becoming repetitive, or saturated. Despite temporal, geographical, and methodological differences of data collection, the secondary data, comparable enough with the primary data, neither yielded influential new codes nor changed our overall theoretical schema; instead, with more additional illustrations, it helped us to articulate some specific concepts, thereby strengthening the clarity of the schema and our sense of data saturation. In retrospect, the comparability of the two data sets is perhaps due, in large part, to the focus group participants’ emphasis on their work-ordered day experiences, the topic of individual interviews in the earlier study. Thus, we primarily present the focus group data here, a few excerpts containing typical themes from the secondary data for supplemental reasons.

## Results

Three focus groups, consisting of twenty-one participants in total, took place at private areas within the Clubhouses during the work-ordered day. In this section, participants’ actual words either have double-quotes or are indented.

### Aspects of everyday participation

Our participants’ narratives concentrated on four aspects of everyday life at Clubhouse (CH) regarding participation, labelled *Coming In, Making Decisions, Doing Work*, and *Locating Oneself in Community*. These, in this order, appeared to loosely coincide with what may be named an everyday participation process, a typical day unfolding in the CH context structured by its work-ordered day program. The scope of our study being the CH context, however, we describe only marginally the Coming In aspect, or the transition from the general community to CH, as an introduction to the three primary aspects, or what we view as the core ingredients of everyday participation. Examination of actions and interactions across these three aspects identified a total of 66 main codes, with two underlying key categories, one concerning freedom to be oneself and the other, connection to others as an equal human being. We have named these two *Autonomy* and *Egalitarian Connection*, seemingly opposite but perhaps related themes, around which we organized our data, as shown in Table I.

**Table I..** Extracted concepts for making decisions, doing work, and locating oneself in community aspects of everyday participation

	Process					
		Properties of Actions/Interactions			Consequences	
Aspects	Actions/Interactions	Autonomy	Egalitarian Connection		Autonomy	Egalitarian Connection
Making Decisions	Expressing Preferences /Ideas Responding to Others’ Input Making One’s Decision Voting	Being Active Initiative Spontaneity Voluntariness Being Eager Feeling Free /Safe to Be Oneself Choose Express Ideas /Preferences Say No Enjoying Process as End	Horizontality Bi- /Multi-Directionality Feeling:Included /Invited Respected /Valued as Equal Human Being Supported in Being Free to Be Oneself Encouraged to Take Risks Trusted in One’s Ability Deciding Together		Empowerment: Augmented Sense of: Power /Control to Influence Ownership Knowledge Purpose Motivation	Group Morale /Camaraderie Augmented Sense of: Togetherness /Fellowship Being Part of /Included
Doing Work	Doing a Task /Things Corresponding w/ Each Other Helping Each Other Learning Skills Working Side by Side	Being Active Spontaneity Voluntariness Initiative Being Busy Enjoying Work as End Strength/Interest-Work Congruency Persistency /Will to Continue /Finish Sense of Purpose Sense of Responsibility	Horizontality Mutuality Collegiality ”Working Together”-ness		Forgetting about Problems “Getting a Life Back” /Healing Augmented Sense of: Confidence /Self-Sufficiency to Do /Accomplish /Make a Difference Motivation /Productivity to Do More Doing “Meaningful‘ /’Valuable” Things Hope /Direction	Sense of Community: Feelings of: Being Included/Part of Being “Needed” /Expected Being Valued /Seen as Asset/Important/Useful /Worthy Being Appreciated Augmented Sense of: Collegiality ”Working Together”-ness Fellowship
Locating Oneself in Community	Socializing Making Friends Helping Each Other Doing Rituals	Being Active Spontaneity Voluntariness Initiative Feeling Free /Safe to Be Oneself Enjoying Being Present as End		Horizontality Bi- /Multi-Directionality Mutuality of Respect/Trust Mutuality of Care /Support Feeling Included/Welcomed Sense of Belonging /Connection /Togetherness Sense of Being Among Equals	Forgetting about Problems “Getting a Life Back” /Healing Sense of “Meaningful Life” Sense of Well-being	Appreciation of Mundane Socialization Anticipating /Trusting “Positive Input” Augmented Sense of Belonging /Togetherness “Getting Along”/“Smiling Faces”/ Laughter /”No Arguments” /”No Fights” Sense of Connection to /Being in a Broader Community

Notes: The three “Aspects” are blended with each other. Each “Aspect” is divided into objective properties (upper rows) and subjective properties (lower rows). Under “Process” and “Consequences” columns, initial bullet points represent codes and indented bullet points represent sub-codes. Double-sided arrows signify a dynamic relationship between autonomy and egalitarian connection. A slash between codes signifies terms that are almost synonymous. Double-quotes signify distinct *in vivo* codes.

Participants typically spoke of participation as, “doing things” and “talking with people.” These two phrases, which we identified during our early analysis, were consistent with dictionary definitions of participation, such as *the process or fact of sharing in an action, sentiment, etc*.; *(now esp.) active involvement in a matter or event, esp. one in which the outcome directly affects those taking part* (OED Online, n.d.), allowing us to assume that participation involves people or social interactions, whether actual or virtual. We further noticed that participants talked about quality participation as signifying “being active” in doing “meaningful things” and talking with “nice” and “friendly” people, which “gets [them] to participate more.” These initial findings generated important elements of our guiding questions (Corbin & Strauss, 2015) as to what they meant by meaningful things and nice or friendly people.

### Coming in

The simple act of coming in and opening the door to a place where a person is going to spend a day can have symbolic meaning. For many of our participants, it seemed to mean entering a world of “positive things” to come, as opposed to staying home, preoccupied with problems. One participant, for instance, anticipated his day opening with “smiling faces”:

When I come through that front door there’s always smiling faces from the employees here. [They] are very encouraging because I come in here sometimes with my head down and as soon as I talk to them I say this could be a good day, why make it a bad day, and it’s just helped me all around I like to um socialize. (Participant 1 [P1], Focus Group 1 [FG1])

Some people come with different expectations. One participant was clear in her purpose: “I like to participate in the work-ordered day.” (P2, FG2) Another person, while appreciating good support from her children at home, said, “I want to have a life outside of [housework].” (P3, FG2) One participant, living alone, was trying to keep her life busy with various activities, including online educational programs: “I don’t feel like participate is quite the right word in a sense. … I’m doing something … but I don’t have any one-to-one interactions with somebody.” (P4, FG3) Still some others come withdrawn or as one participant put it, “catatonic,” only to sit in a corner, but they do come all the way from home.

### Making decisions

#### Unit Decision-Making

Of several kinds of decision-making (DM) activities discerned in our data, participants’ accounts centred on those made as part of the routine work unit meetings. Two aspects of these unit decision making (UDM) were identified, first decisions involved in planning the work-ordered day, setting out the necessary tasks and identifying volunteers for each task needed to run the CH. Second, after dealing with the everyday routine, the meeting moves on to planning special activities, such as regular or occasional social events. The latter entails more decisions, including what, when, where, who, and how, more overtly using decision-making rules including voting when necessary to reach group consensus. Both UDMs appeared not only to facilitate empowering the individual and the unit—processes themselves which members appeared to enjoy more than a step to a work action—but also, as exemplified later, to generate prototypical patterns that permeate the work-ordered day.

The UDM processes described by our participants revealed an autonomous nature of their involvement and an egalitarian nature of the milieu. To illustrate, first, the physical environment, typically decorated with flowers and drawings on the walls, is inviting. Each unit is equipped with a white board, on which small tasks are organized such that everybody can see available activity options needed for running the CH. The UDM meeting begins with greetings and general invitation to activities. A staff member may make verbal announcements, informing members of opportunities. Individual members then will know: “something new … what you have to do in the future work … a job, business, and what we’re going to do for the house … cleaning stuff, you know, … for today.” (Secondary Data [SD] 1) The staff also may clarify purposes and benefits of work-ordered day activities, while conveying with care their expectations of members to volunteer for tasks:

We want you to do some work. We don’t want you to feel alone … we want you because you stay there [and] you have nothing to do. We want you to do something. … Just to work on it for a half hour or for 20 minutes … . When you finish everything then you come and it’s ok. … We want you to work, you know, because you stay alone we have some work to do and we can leave you alone, you know. We want to give some you have to do something. … We want you to be active. We want you. (SD 1)

UDM as a form of group decision-making assumed dual processes of individual and collective DMs. For the former, almost all participants explicitly or implicitly appreciated that the environment respects their individual choice: “Well, I mean, seems like you’re free to volunteer here like you’re not being pressured I guess … and like when I’m not pressured I think I respond better.” (P6, FG1) One participant said, “The staff *tell[s] me* what I *have t*o do” (SD 1, Italicized by authors), but this did not mean *forces her*: “No, no, I don’t feel coerced.” (SD 1) It meant her own will to cope with her depression by doing; coming in the CH feeling down, but being encouraged by the invitation, she meant *pushing herself* to do the work because she understood or experienced the benefits of doing it:

Because when you’re depressed you know brain, you don’t feel good. When I get some work to do, you work for your brain you know. It works for your body you feel ok after you’re busy doing something … . At home, you feel alone. And when you come into program, you have something to do. You know you feel uh more active. Yea. (SD 1)

Some individuals may end up choosing not to do anything; yet, as they stated, they made this choice without feeling blamed or ostracized. They instead seemed to feel their choice was respected and understood, whereby, as they recounted, even those who were self-absorbed and unresponsive when they first came to CH eventually joined in: “When I started taking the skeleton out of [the] closet that’s when my healing came. … I was seeing people over there I was like I want to smile with them okay I want to joke with them.” (P5, FG1)

Participants also appreciated the equal opportunity and support for giving one’s input: “Everybody’s being chosen as the same type of person.” (P7, FG2) or “We’re equally here with each other.” (P7, FG2)

I am supported in a way that I feel good about. The important thing was that I felt that I was being equal … some kind of opportunity to … participate in the decision making process … they have freedom of, like choosing the thing that they want to do and no one is holding your hand in the sense that … you can make your own decisions … I am surprised that we had … so much power over deciding. For example, … “foods that are on the menu but we can change them, we can modify them, we can make them our own if we want and if … there was a problem we were encouraged to solve the problem ourselves of course being able to ask but nobody was like being a nanny for us yea … . For example, … I have been able to make most of the vegetarian menus vegan if I like so there is no meat or dairy products … I didn’t want there was cheese in this … I decided it in a group while we were working and it was okay that I asked that to people who run the [kitchen] and it was okay and we were encouraged to go ahead … so they trusted me being able to … do my own decisions.” (SD 2)

How freely one can say no may be a useful indicator of how deeply the spirit of individual choice is embedded in the environment, not just during UDM but also permeating throughout the work-ordered day. Some members more than others did seem to struggle at first about their rights to say no, but then realized it is allowed or supported to practice it and gradually come to terms with being themselves who say no. One member’s narrative, for example, portrays this:

When I first started here I was in the clerical unit … doing morning tasks and afternoon tasks, and then I decided I wanted to go upstairs and cook a little bit, so I stayed up here for about 6 months … they had me making biscuits and … fried chicken and … doing all kinds of stuff upstairs that I really like … them enjoying my food, … [a staff downstairs] tried to keep me come back [laughs] keep me coming downstairs, because he said he needed me downstairs. I said no sir, thanks. (P3, FG2)

Autonomous DM and its egalitarian environment were evident also in UDM as collective process. First, “one person does not make decisions … we make it as a group.” (P9, FG2) Second, the process unfolds in a multidirectional fashion—by individuals expressing ideas, preferences, or opinions, and others then responding with appreciation to each idea, preference, or opinion that is shared. The group further exchanges and discusses the shared materials, and, once saturation is felt, individuals and the unit make their final decisions on what to do. A participant illustrated such an interactive process: “we brainstorm … we vote, … people try to bring their opinion” (SD 1), whereby the unit comes to share the decision: “What we have our plan, is … not personal … something for everybody.” (SD 1) Narratives indicated how the process occurs in a milieu that allows members to freely express their ideas or opinions (“every member … they bring up … ideas” [P10, FG2]) and how adequate time (“40 or 50 minutes” [SD 1]) is taken to reach a general consensus: “We all vote for something … until we have a consensus.” (P2, FG2)

A sense of ownership and enhanced group morale seemed to be the end of the UDM process even though some lose over “the majority [who] wins” (P11, FG2): “All the decisions are made through the members.” (P9, FG2) Note that decisions are not made solely by members; they are somewhat circumscribed in the sense that staff are normally present during UDMs and certain aspects of decisions, such as budgetary decisions, are exclusively under staff’ control. As some stated, staff are responsible for final decisions. Participants appeared, however, to regard it as the staff’s role, which did not seem to interfere with including the staff as part of the collective “we:” “All of us make the decision together.” (P9, FG2) Our data did not suggest that participants were experiencing staff as if they were imposing decisions. On the contrary, with their sense of ownership and togetherness, the unit seemed ready to do the work with their shared purpose and direction: “Everybody knows what to do.” (P9, FG2)

#### Other decision-making

Somewhat marginally, other DM patterns revealed different qualities from those hitherto presented. They seemed to represent, however, other dimensions of our extracted properties, only to validate how these properties are relevant to participation. First, administrative or committee DM opportunities, which are made on organizational or program matters, did not appear to be perceived as open to all members. As one participant indicated, members are not only vaguely informed of these opportunities but also the staff decides who attends:

Basically how it’s set up is members are supposed to be involved with all the staff in everything, I don’t think a lot of members are aware of that so not a lot of members get the opportunities but you’re supposed to be involved with everything, even the away-day they asked the members to come in for the away-days as well. I’ve been quite lucky, but I think that’s because I’ve been coming in and doing quite a lot of stuff that I get picked to go and do that. (P12, FG3)

As such, the sense of ownership did not seem to be the case with this DM type, as reflected in a member’s use of language, “staff make *their own* decisions … participating in some of the *staff’s* events” (P9, FG2; Italicized by authors). Interestingly, though, our participants’ tone was not necessarily negative; to the contrary, most appeared to take for granted or respect staff-only decisions. They seemed to understand and rely on the policy and rules as well as staff guidance, which could be viewed as a manifestation of trust in the staff or CH in general.

If we have … difficult decisions … we cannot make up our minds [by] our own selves, and the rules at CH … our policy, our Standards we’re supposed to go by here at CH … we go to our head leaders, they help … in that matter. (P7, FG2)

There were some negative cases. One participant, for instance, who missed UDM meetings for a few months, stated, “I think not being in unit meetings meant that I felt that I missed out on a lot of shared information about opportunities and things to get involved with here.” (P4, FG3) By not being seen by others, the participant appeared to be lost in the unit, or left out from the teamwork that followed. Another negative scenario concerned CH-wide policy meetings, against which the same participant commented their inadequate information sharing and top-down style: A staff member there presented “text-heavy” slides that were hard to follow and kept “telling people about something … [and] decision-making [was] not shared with members.” (P4, FG3) This participant concluded, “it feels like kind of discussion is kind of shut down if it veers from what the management wants.” (P4, FG3) The meeting did not sound very inviting or encouraging, let alone like there is a sense of ownership over the decisions being made. These DM experiences certainly account for the importance of an egalitarian context.

### Doing work

Common elements of work include self-sufficiency or independence. The work-ordered day, however, designed to work in teams to operate the CH, involves work collaboration among people, which may make the work go above and beyond self-sufficiency. While participants appreciated being busy in doing things, instead of “sitting all day” at home, they typically went on to tell how work in relational contexts made it more meaningful. Below we first focus on Doing Work for independence; then we describe how relationship might enrich the work and the enriched work experience might in turn not only augment the motivation to do more but also enrich one’s social world, seeing oneself as part of a community, or Gemeinschaft of sorts.

First, most participants voiced their appreciation that there are “always things to do.” These things are usually designed to be small enough to be manageable to avoid imposing an undue workload. Participants indicated that focusing on doing these tasks helps them stop “thinking about problems” all the time and to gain or regain confidence thereby feeling productive, to do more and to get “a life back.” Meanwhile, narratives also showed that the match between a thing to do and one’s strength or interest is critical for the individual to enjoy the work as an end or to be persistent in meeting a challenge. Computer work, for example, may work well for some people, but not for others. One participant expressed her dissatisfaction with a lack of opportunities for craft enterprise as her “employment avenue.” For this member, the computer gave her “something to do,” but as it was not her interest, it was less meaningful for her, which discouraged her from continuing. On the other hand, when opportunities matched their strengths and curiosity, participants expressed their enthusiasm about doing things, saying they were used to, good at, or knowledgeable about them. One said, for example, “I love working on computers. … I had some knowledge of the computer so.” (P13, FG1) Other members sounded excited about learning various life skills, such as cooking or healthy lifestyle, and their growing confidence to become self-sufficient, which in turn encouraged them to do more:

I’ve um learned a lot about cooking that I didn’t know and … it’s very beneficial because one day I might be alone. I live with my mother and she cooks but she’s gonna be gone to [AB] for the next two and a half weeks, so I gotta cook on my own, so that’s gonna be the real true test [laughs] how good of a cook am I. So for the past three months … I’ve been cooking Mondays, Wednesdays, and Fridays in the afternoons. So I’ve learned a lot in three month’s time. (P14, FG2)

Hope seemed to be a consequence and reinforcement of doing work. One member was enjoying her work, feeling good about her social skills, whereby hope for a job emerged:

I enjoy the telephones, and I’d say hello this is [P7] … this is CH [P7] speaking, how can I help you? You know that I’m helping somebody you know to communicate with a member of staff they didn’t want to talk to, you know, I ended up enjoying that, and I thought about getting a job as a receptionist. (P7, FG2)

A young participant, who had dropped out of school before she came to CH, was persistent in restoring her self-sufficiency in work skills. While reminding her, “You need to … graduate,” she was also holding onto the hope that she might be able to obtain employment, which seems to have been steering her to do more:

I work in the clerical unit most of the time, doing the telephones and desks, computer tops and cleaning, and things like that, and you know that you’re going to get those skills back again and get your own purse again. (P7, FG2)

#### Working together

“We all got, you know, to know each other and it’s been a good experience. … And I love being a part I did.” (P15, FG1) Narratives indicated participants felt more active or productive in collective or relational contexts that entail mutual and horizontal interactions. Often expressed as “working together,” they recounted that work feels more “meaningful” or “valuable” because it involves feelings of the individual being “needed,” appreciated, “useful,” and connected with work colleagues, as elucidated below:

As in any society, novice members at first receive support to varying degrees for work skills and learn from experienced team members. In this relational context, they gradually gain confidence to do things on their own while learning to help others in the same way they were helped. One participant recalled, “I didn’t really know what I was doing at first but [a staff] helped me out along the way and everything and I started doing it on my own.” (P5, FG1) Members like this then take the initiative to help others:

One of the things that I don’t do is wash dishes. But if I see there’s only a little bit of people in the kitchen and I see there’s maybe only 2 or 3 other people in the kitchen, they need help doing something else, I’d step in and help them out. (P13, FG1)

Members not only help their fellow members but the staff as well. The staff and members also work literally side by side, mutually “helping each other” as equals, versus a top-down relationship:

I’ve been doing different activities and helping out with different things with [a staff] and [the staff] would help me do presentations at [a program] and go to nine meetings and helped out there. At [a CH-affiliated program] I taught members, different people, how to use a computer. (P13, FG1)

Expressed as central to the meaningfulness of doing things was their sense of being useful and important, or self-worth:

Well. I found interesting things to do, like making our magazine and writing texts and making layouts, and dealing with photographs, things like that … . But mostly I think it was because, I felt I was seen asset, valuable person. It makes me feel kind of special. … I think it does real things, someone smiling at you and say how good you are. … That’s good. (SD 3)

Notably, narratives often indicated some turning point in this context—the meaningful work experience appears to begin, in turn, to go deeper, transforming, or strengthening and expanding, one’s social world. As stated by the above participant, “I guess, it’s, I have a need to be loved, something community. Like something meaningful in my life and socializing.” (SD 3) Another participant simply states, “My participating at CH felt like I was needed in the world.” (P16, FG2) Yet another participant was more descriptive, “I learnt a lot about cooking, learning to … talk to other people that’s dealing with the same thing I’m dealing with through life. I mean … it’s been a great experience working being here.” (P15, FG1)

### Locating oneself in community

Group work, by its nature, mobilizes communications among people to achieve a shared goal. Out of necessity, people “correspond with each other” (P9, FG2), such as discussing task-divisions, asking for help, or offering help. Often, people also may make casual conversations between work tasks. One member said, “Normally you’ll see me, when you walk through the door, I’m a piece of the furniture on reception basically, everyone, when they first meet me, they go, oh, I’ve met you on reception.” (P12, FG3) Another member took a break, started chatting with a member he was working with, and both wound up going out for a movie after work. Likewise, through work-mediated interactions, people become familiar to each other over time, spontaneously initiating various levels of everyday socialization or making friends—dynamic processes which may begin to form a community that has, as one participant articulated, “something which we share, which we have in common, … basic humanity, basic feeling of person to person interaction.” (SD 2) This sense of community in the CH was omnipresent in our data:

It has done me a world of good to socialize to help run the desk downstairs I know its hectic and things get on my nerves but then I love the people here they’re friendly and there’s always something to do. It’s a good day program … to be in, it gets me out more to participate more and be with friends and people that care. I mean community. Yeah. (P17, FG1)

Quality communal participation involved actions and interactions such as socializing, making friends, helping each other, characterized by horizontality or equality among people, bi- or multi-directionality of communication, mutuality of respect, trust, care and support, and sense of belonging. We see this as a type of egalitarian community.

To illustrate, many participants expressed appreciation for the healing power of seemingly insignificant mundane socialization such as “how’re you doing,?” “just having a conversation” (P18, FG3), or even just “hello,” or “smiling faces.” Some find their place simply in being among people while others bring jokes: “I love to make people laugh because that’s what CH gave to me.” (P5, FG1) Still others find valuable opportunities for making friends: “it has helped my mental health to socialize with people and to have friends.” (P10, FG2) The participant, who had never had any friends at a young age, was appreciative of her discovery: “I realized I could make friends here.” (P10, FG2)

Participants’ narratives indicated the egalitarian milieu of the CH community in various ways. A number of participants used non-specific pronouns: “And it’s all those smiling faces [and] encouragement, how’re you doing and if *they* feel you’re having a bad day *somebody* might come up to you and give you a positive input” (P1, FG1; Italicized by authors). Whom this participant meant by “they” or “somebody” became the kind of questions we sometimes posed in our interviews, to find it coming from both staff and members, giving care and support in the same way—an egalitarian tradition or culture, which makes it difficult for a visitor to distinguish between staff or member at first sight. To wit, both parties are intermingled, helping each other side by side. While participants appreciated that the staff remains to make themselves available for support and care as needed (“You could call the staff to the side and talk to them about anything.” [P3, FG2] “They [took] me to the gym, I got a NPR worker.” [P15, FG1]), particularly guidance and encouragement (“They encouraged me to go to the gym.” [P7, FG2]), they also seemed pleased with their transformed, horizontal, or egalitarian, relationship with staff that transcended the work relationship. Members come to enjoy casual socialization between work activities. Below members joke around with the staff in friend-like closeness:

[P5:] [Director is] very engaged. [P15:] He is. [P13:] … he’ll see how everybody’s doing, sometimes if … no staff in there with you, he’ll come in there and he’ll … help do. He’ll help out with the dishes there. [P15:] Yeah. [P5:] If you’re not wearing the right things … [P19:] You like it though. [P13:] Ooh. [Many:] Ha Ha. [P5:] He’d be, like, you shouldn’t wear that. One day he told me he said your hair looks bad, I said I know I know, man, I know. [P19:] Yeah, cuz sometimes he’ll tell me what are you looking at, I’d say what are you looking at. [Many:] Ha Ha. (P5/13/15/19, FG1)

One participant described how he might reach out to their fellow members in need. This may speak to an almost instinctive depth of sensitivity to the suffering of the other, which perhaps only the person who experiences the same may understand:

Everybody feels comfortable, nobody has to be shy or uneasy around everybody because we’ve been here so long. Everybody knows how to take each other, you know, if you’re in bad mood that day everybody knows how to give you your space, and … let you work it out all in your own, or if you want to talk to one of us, you know, you’ll come, pull one of us to the side. ‘Sir, I need to talk to you about something you know.’ (P9, FG2)

Participants also highlighted the intrinsic reward of helping others:

Like I said, listen to their stories, and I’m telling mine, and I forget about mines and listen to theirs, and … it’s been a great support coming here, and taking … um, it taught me to keep the faith, and realize what I was going through somebody else was going through something worse than I was. (P3, FG2)

The reward can be much more powerful, involving “a sense of real satisfaction.” (P12, FG3)

Yeah, I suppose what I like about it is I might come here all sick and depressed in the morning but as the day goes on, I get to meet people and helping their situation and then I’m feeling more upbeat and better in myself from helping people. (P12, FG3)

Overall, the community described above seemed to feel to many like “We are a big family” (P9, FG2) and a sense of belonging was ubiquitous across narratives. Those bonds may surface when familiar everyday life encounters unexpected disruptions, for instance, when someone is “away on a weekend or social”: “We’re always thinking about each other, how’s this person doing how that person’s doing, you know, … we’re always thinking about what’s coming, we’ll be back next week.” (P9, FG2) Or, when someone dies:

Every now and then we’ll have a member to pass and we all feel that because we don’t see that member in the building no more, and it takes an effect on all of us when one of us pass away or something, you know … the loss everybody feels it because the person is no longer here and we miss that person so much, you know, that, you know, it really takes. … it takes an effect on all of us, you feel it. (P9, FG2)

Personal bonds may be challenged when conflict occurs among community members. Our participants, however, while openly talking about disagreements happening at times, indicated that there were “never any arguments … no fighting here.” (P2, FG2) If the problem is difficult, they may ask staff for help to solve it through two-way communication and, eventually, “We still come back together … and talk to each other.” (P13, FG1) In short, people here “get along with each other.”

#### There is no outside

One source of speculation that arose was whether the sense of belonging to the CH community could only develop through both the egalitarian member-to-staff relationship and member-to-member relationship. A few negative cases can give some validation for this. Three participants, still relatively new to CH, expressed their rather distant relationships with the staff. One had never had even a single conversation with her designated staff worker since she came to CH. This participant appeared to be confused about what CH was. The other two perceived that support from the staff was absent when things were not going well. One of them indicated her disappointment because she had a problem with other members in group-work situations, which in turn hampered her relationship with the staff because the staff was unable to offer any useful “guidance” for handling the challenge. Both participants indicated they were lost at the moment, and one of them had stopped coming to CH for a few months. The other had voiced the need for improvement but was “brushed off.”

Some members may experience a sense of belonging only to staff or peer sub-community, but not to the whole CH community. We further postulate that those who experience the sense of community to the whole CH community are more likely to transfer this experience into the larger society as well, whereas those who experience the sense of belonging only to a sub-community may not make the same kind of transfer. One participant, for instance, had positive relationships with both staff and members, comparing CH with a family. He responded promptly to our probing: “Outside of CH? There is no outside CH. We always go out together.” (P9, FG2) Perhaps they are right. With the wall between inside and outside having melted down, they may very well be part of, or connected to, the broader community through the CH lifeline as their home base. Their everyday life overall appears to feel “[getting] back out in the world” (P7, FG2), different than it was before they first came to CH as an “outcast.”

On the other hand, another participant who expressed that she had a close tie to the member sub-community did not sound happy about the member-staff relationship even though she was grateful for the staff giving her lots of opportunities. She felt “invisible” with “no support” provided by the staff when she was feeling ill: “I don’t get the support here when I’m not well.” (P12, FG3) She said that support in times of need “always come from members,” while identifying the staff with conventional mental health professionals *outside* of CH, who she had experienced as “terrible” at times. Her sense of belonging to CH sounded somewhat compromised: “Where would I be if I didn’t have CH? So it’s doing something to support me.” (P12, FG3) Her immediate response to our probe about her relationship to the broader community was, “I feel like I only socialize at CH, I haven’t got the confidence yet to go to build relationships out of here,” only to add later, “I socialize *outside* CH with CH because we have our socials that we go to. … So I do that *outside* but not separate from CH if that makes sense.” (P12, FG3; Italicized by authors) The tone suggested that a wall of sorts exists between the inside, or her circle of peers, and the outside, the clinical side that includes CH staff.

## Summary and Discussion

The aim of this exploratory study was to conceptualize participation in the CMH context from the perspective of service users, focusing on the mental health Clubhouse context as an example.

Our participants’ narratives clustered around four aspects of what we named everyday participation process in Clubhouse life: Coming In, Making Decisions, Doing Work, and Locating Oneself in Community, with the last three the core. These three appeared to be blended throughout a day at Clubhouse rather than forming clear-cut stages. Beneath everyday participation, we identified two themes, namely Autonomy and Egalitarian Connection. The Autonomy theme subsumed properties characterizing quality participation such as being active, being free to be oneself, persistence or will to continue, enjoying activity (including decision-making) as an end, and sense of power to make a difference, ownership, confidence to achieve, and getting a life back. The Egalitarian Connection theme encompassed properties such as horizontality and bi- or multi-directionality of interactions, mutuality of respect, trust, care, and support, feeling included or invited, feeling valued as an equal human being and useful as well as sense of togetherness and community belonging. At the end of the day, life appeared to feel more positive and meaningful, motivating the individual to come back to the Clubhouse the next day.

Our data further suggested that Autonomy and Egalitarian Connection have a dynamic relationship. It appeared that participation loses its quality in a top-down or paternalistic relational context, which can undermine Autonomy and, in turn, can undermine one’s sense of connection to the immediate environment one interacts with, thereby closing oneself off from the world. Conversely, participation gains its quality, or meaningfulness, when an egalitarian relational context augments one’s sense of autonomy, a positive experience which in turn enhances a sense of connection to an autonomy-supportive egalitarian environment, thereby strengthening and expanding one’s egalitarian social world. The augmented sense of Autonomy and Egalitarian Connection, in turn, sets the stage for Coming In the next day to do and socialize, or participate, more.

In brief, we have come up with a 3 × 2 axial model of everyday participation:

Proposition 1: Participation denotes a behaviour comprised of three activity domains, Decision-Making, Activity Proper (e.g., Doing Work), and Locating Oneself in Community.

Proposition 2a: Participation signifies actions and interactions that concern Autonomy (e.g., choice, power, means-ends congruence, freedom to be oneself) and Egalitarian Connection (e.g., being valued as equal human being, mutual respect, support, and care, sense of togetherness).

Proposition 2b: Autonomy and Egalitarian Connection have a dynamic influence on each other, generating a movement towards another round of meaningful participation the next day.

The present study adds to the literature with inductive knowledge grounded in the first-person level of data and can provide a framework for understanding participation in the domain of community mental health practice. Overall, our findings are consistent with the Clubhouse literature as well as the general theoretical literature on participation. First, themes we identified largely agree with those described in the Clubhouse program literature including the Standards (Anderson, 1999; Beard et al., 1982; Clubhouse International, 2021; Doyle et al., 2013; Hänninen, 2012) as well as themes identified in the research literature (Carolan et al., 2011; Hancock et al., 2015; Herman et al., 2005; Kinn et al., 2018; Mutschler et al., 2018; Norman, 2006; F. P. Chen & Oh, 2019; Pardi & Willis, 2018; Raeburn et al., 2013, 2015; Rice et al., 2020; Schiff et al., 2008; Tan et al., 2018; Tanaka et al., 2015; Tanaka & Davidson, 2015a, 2015b). Our study focus being service users’ perspectives, it is notable that the Autonomy and Egalitarian Connection themes we found corroborate staff views on what fosters member participation (F. P. Chen & Oh, 2019).

By and large, our findings also support participation theories and conceptualizations existing in the general macro domains, formulated through deductive approaches (Arnstein, 1969; S. C. Chen & Raab, 2017; Charles & DeMaio, 1993; Cohen & Uphoff, 2011; Cornwall, 2008; Ginis et al., 2017; Jenkins, 2009; Kelty et al., 2015; Law, 2002; May, 2006; Pateman, 1970; Pretty, 1995; Stage & Ingerslev, 2015; Tritter & McCallum, 2006; White, 2011; WHO, 1991). At the same time, our findings regarding the bi-axial component spanning autonomy and connection provide greater support for the literature that speak to the multifinality (Leighninger, 1977) of participation (Ginis et al., 2017; Jenkins, 2009; Kelty et al., 2015; Stage & Ingerslev, 2015; Thomas, 1994; Yilmaz et al., 2008, 2009), as opposed to the traditional, uni-axial line of work (Arnstein, 1969; Carpentier, 2011; Pateman, 1970; Pretty, 1995), whose ultimate concern tends to be decision-making and power. Our model of participation further aligns with philosophical principles (Buber, 2012; DeLanda, 2009; Freire, 2000) put forth by Thomas (1994) and Stage and Ingerslev (2015), maintaining its dynamic aspect in that autonomy and egalitarian connection may not be orthogonal, but rather, conditioning each other, forging internally (Buber, 2012; Freire, 2000) and externally (DeLanda, 2009), a movement towards expansion of one’s social world. These formulations dealing with autonomy and egalitarian connection, in fact, are relevant to the population under study, mapped onto personal histories of many individuals enduring paternalistic oppression and social exclusion (McLean, 1995).

Interestingly, our autonomy-connection conceptualization also fits well with a line of Aristotelian thought (Aristotle, 2009, 2020; Fowers, 2012; Young, 2017), which places self-sufficiency and pleasure (means-ends unity vs. separation) and friendship (individual vs. shared benefits) under the purview of quality of life, or “eudaemonia.” Although, as DeLanda (2009) points out, the Aristotelian view may not explain the dynamic relationship between autonomy and connection, it can provide a useful framework for our formulation connecting Autonomy and Egalitarian Connection to well-being. Our formulation also is consonant with Aristotle’s idea of democracy, which is grounded in an egalitarian ethos, as exemplified by “the notion that those who are equal in any respect are equal in all respects; because [human beings] are equally free, they claim to be absolutely equal” (Aristotle, 2020, p. 9). In this respect, participation, which concerns autonomy and egalitarian connection, points to quality of life, or well-being.

While we are confident in reasonable conceptual saturation for our study purpose given the triangulated, large, and in-depth data collected mostly by the authors themselves of a shared professional identity, or research team as instrument (which, however, excluding perspectives of other professions, also presents an inherent limitation), the study has limitations that necessitate additional theoretical sampling. First, the study did not fully address conditions such as structural or personal conditions affecting participation processes, thus our model is restricted in its comprehensiveness. Second, our model is incomplete because the scope of this study was the Clubhouse context. Although we assert that our 3 × 2 model has value as the core of what we may conceive as a complete model in our future study, we need more data on participation in the general community to fully capture it, including for example, its Coming In aspect. Third, generalizability is limited at various levels. Besides the limitation deriving from purposive sampling, the model is based mostly on components of the Clubhouse work-ordered day. Intuitively, while decision-making is always part of any activity involving participation—a necessary component of the model—voluntary work is not. Future studies should compare and contrast our findings with data on other types of activities, including employment, educational, and recreational programs in various CMH contexts. Finally, our current data are confined to those in Western democracies. Data from countries that have other cultural and political underpinnings may reveal different conceptualizations of participation or, at least, add variations to properties extracted in this study.

Despite the limitations, the grounded theory we discovered in this study can provide useful implications for practice. It gives a sense of direction for our everyday practices, reminding us that participation is a dynamic process between autonomy and relationship towards well-being—that the act of doing or talking represents the autonomous self in an egalitarian environment, whether in a dyad, small group, or community context, whereby the actor learns to strengthen and expand their egalitarian world. Particularly notable is the importance of egalitarian practitioner-service user relationship, which may easily fall into an asymmetric or paternalistic one. Training should help practitioners ingrain egalitarian relationship skills that promote an egalitarian environment, which appears critical for users’ active and meaningful participation.

### Conclusions

The study explored a conceptualization of participation in community-based mental health contexts from a first-person perspective, using the Clubhouse model as an example. Our embryonic grounded theory suggested: Participation denotes a behaviour (1) comprised of three activity domains—decision-making, activity proper (e.g., doing work), and locating oneself in community; (2a) entailing actions and interactions that concern autonomy and egalitarian connection; (2b) which, dynamically, possibly synergistically, reinforce meaningful participation the next day. Themes identified corroborate those found in the Clubhouse literature. Our conceptualization, while generally consistent with participation theories long existing in other domains of study, is more in line with work that has brought the connection theme equally to the forefront. Our findings suggest that training for mental health practitioners focus on egalitarian relationship skill development to support service users’ meaningful participation and getting-a-life-back experiences, ultimately their well-being, or meaningful life.
